# Properties of Concretes Incorporating Recycling Waste and Corrosion Susceptibility of Reinforcing Steel Bars

**DOI:** 10.3390/ma14102638

**Published:** 2021-05-18

**Authors:** Zinoviy Blikharskyy, Khrystyna Sobol, Taras Markiv, Jacek Selejdak

**Affiliations:** 1 Faculty of Civil Engineering, Czestochowa University of Technology, 69 Str. Dabrowskiego, 42-201 Czestochowa, Poland; zinoviy.blikharskyy@pcz.pl; 2 Lviv Polytechnic National University, Bandera Str. 12, 79000 Lviv, Ukraine; khrystynasobol@ukr.net (K.S.); taras.y.markiv@lpnu.ua (T.M.)

**Keywords:** Portland cement, ground granulated blast furnace slag, fly ash, pozzolanic reaction, corrosion, rebar

## Abstract

In this paper, properties of concretes incorporating recycling waste and corrosion susceptibility of reinforcing steel bars were studied. It was established that fineness of ground granulated blast furnace slag (GGBFS) and fly ash (FA) and their simultaneous combination have an influence on the kinetics of strength development of Portland cements and concretes. The compressive strength of concrete containing 10% by mass of GGBFS and 10% by mass of FA even exceeds the compressive strength of control concrete by 6.5% and concrete containing 20% by mass of GGBFS by 8.8% after 56 days of hardening. The formation of the extra amount of ettringite, calcium hydrosilicates as well as hydroaluminosilicates causes tightening of a cement matrix of concrete, reducing its water absorption, and improving its resistance to freezing and thawing damage.

## 1. Introduction

Environmental problems are becoming more and more serious from year to year. Despite the remarkable development of scientific and technological progress, some industrial processes result in the significant production of waste, which cannot be landfilled completely and present a big problem for both the producer and the environment [1]. The waste accumulation is observed every year all over the world. Most of these industrial wastes are by-products such as granulated blast furnace slag and fly ash. The building material industry is one of the areas where such wastes can be utilized in large quantities.

The cementing materials and products on their basis have played a very important role in the development of construction for many years. The Portland cements and concretes are the most used materials in the construction industry [2,3]. The technology of Portland cement production, which remains the main component of concrete on the basis of a mineral binder, causes significant greenhouse gases emission, which results in global warming and climate change [4,5]. The cement production process generates almost 7% of the global greenhouse gas emissions [6,7,8,9,10]. Using waste as supplementary cementitious materials (SCMs) instead of clinker in the technology of cement production and cement in concrete will contribute to the partial solution of the above-mentioned problems [11,12,13]. Ground granulated blast-furnace slag and fly ash are among the types of waste that are utilized successfully in the construction industry and are attributed to the supplementary cementitious materials.

GGBFS is rather valuable waste and can be recycled in environment processes, agriculture, and the construction industry. Ground granulated blast furnace slag has begun to be used widely since the discovery of its latent hydraulic properties. Many researchers have been conducting lots of experiments to determine the properties of cements and concretes containing GGBFS and fly ash [14,15,16,17,18,19,20,21]. According to Ivashchyshyn et al. [15] and Kurdowski [22], properties of cements and concretes containing blast-furnace slag depend on the chemical composition, fineness of slag, and the amount of vitreous structure in slag. Kumar et al. [23] and Zhu et al. [21] also concluded that the efficiency of slag depends on its particle size. Pal et al. [17] indicated that the fineness of slag and slag/cement ratio affect the strength of concrete incorporating GGBFS. Osborne [24] confirmed many benefits of the effective use of GGBFS in concretes such as reduced heat evolution, lower permeability, higher strength at later ages, and increased corrosion resistance. It was also pointed out that the use of very high levels of Portland cement replacement with slag can influence the corrosion susceptibility of reinforcing steel. Menéndez et al. [25] proved the efficiency of ternary blended cement incorporating limestone filler (LF) and blast-furnace slag. LF in such systems causes an increase of hydration at early stages while slag contributes to hydration at medium and later stages. Domenico et al. [26] studied the behavior of reinforced concrete (RC) beams containing electric arc furnace (EAF) slag under the load. EAF slag was used as a coarse aggregate. It was concluded that ultimate flexural and shear capacity of the above-mentioned beams was higher than the corresponding traditional RC beams, due to the improved properties of EAF concrete.

FA is a by-product of electricity generating plants. It is used both in the technology of cement and concrete production [20,27]. It has positive influence on the properties of fresh and hardened concretes such as workability, strength, drying shrinkage, thermal properties, and abrasion resistance [28,29]. Ghais et al. [30] state that the addition of 10% by mass of fly ash increases the concrete strength while the higher amount reduces it. The above-mentioned research was focused on the utilization both ground and unground (unactivated mechanically) fly ash. The usage of unactivated fly ash makes it possible to avoid the extra operations such as its grinding, which reduces the consumption of electricity and, as a result, natural energy resources, which are still used for electricity production, and greenhouse gas emissions. Unactivated fly ash allows for the particle size distribution to be optimized in cements and concretes because it plays a role as a mineral addition with pozzolanic properties and as a microfiller [29].

Sanytsky et al. [20] and Li et al. [31] concluded that the combined use of GGBFS and FA in both cement and concrete is very effective. On one hand, some researchers have reported that the binding system containing Portland cement, GGBFS, and FA provided higher strength at all ages than the ones incorporating GGBFS and FA separately [31,32]. On the other hand, Jeong et al. [33] did not find any differences in the cement system incorporating GGBFS and FA.

Fernández et al. [34] also pointed out that the synergistic effect between GBFS and CFA depends on the chemical composition of Portland cement in the cementitious system, which consists of these constituents. Thus, chemical compositions and the fineness of Portland cement, ground granulated blast-furnace slag, and fly ash can have an influence on the properties of the cementitious system.

The use of GGBFS and FA may also have an influence on the initial pH of the cementitious system and the protective passive layer, which is formed on the reinforcing steel bars due to the alkaline environment in concrete [35]. In the last few decades, corrosion of rebars in reinforced concrete structures is one of the main factors, which causes their extensive premature deterioration and has a serious effect on the serviceability and safety [36,37]. However, there have not been many publications in the literature related to the effect of the amount of SCMs on the corrosion susceptibility of reinforcing steel bars in concretes, in spite of the tendency of the significant reduction of clinker factor (CF) in cements to follow their contemporary tendencies. It can have an influence on the stability of steel reinforcement bars in such concretes. Moreover, it is very difficult to obtain a high strength class of cements with low CF and it can result in the increase in cement consumption in a concrete, cause higher creep and shrinkage deformation and, as a result, influence the durability. The development of cement with a lower substitution level of clinker and a higher compressive strength provides a reduction of its consumption in concretes and an improvement in the mechanical and durability properties of concretes as well as the stability of steel reinforcement bars in reinforced concretes.

According to Yeau and Kim [38], the corrosion probability of reinforcing steel bars in Type V cement concrete was higher than in Type I cement concrete. It was concluded that the corroded surface area depends on the amount of GGBFS replaced and the resistance to steel corrosion is better in Type I cement concrete with higher amount of GGBFS. Topçu and Boğa [39] studied the corrosion performance of steel reinforcing bars in concrete incorporating 25 and 50% by mass of GGBFS. This was done in C1 (uncontrolled relative humidity and temperatures) and C2 (in water with 20 ± 2 °C temperature) curing conditions and at ages of 28 as well as 90 days. The researchers concluded that the deterioration occurrence times extend in concrete containing 25% by mass of GGBFS for both curing conditions. The half-cell potentials of concretes incorporating 25% by mass of GGBFS, which were exposed to C2 curing conditions, have not passed through the area of the possible corrosion unlike other series that have passed through this area. According to Song and Saraswathy [40], the reduction in the pH of concrete containing GGBFS had no significant effect on the corrosion resistance of embedded reinforcement. The researchers also observed that the steel reinforcement bar embedded in cement that incorporated unactivated fly ash suffered severe corrosion [41]. However, concrete incorporating activated fly ash improved corrosion-resistance properties. Polder [42] summarized that replacement of the clinker with 50–70% by mass of GGBFS and 20–30% by mass of fly ash resulted in a higher chloride penetration resistance and electrical resistivity, decreasing the risk of corrosion in environments contaminated with chloride. Composite cements containing 25% by mass of both slag and fly ash behave similarly. Thus, the literature review shows that in some cases, contradictory results in experimental studies have been obtained.

This is why the aim of this research was to establish the effect of fineness of GGBFS and unactivated fly ash on the strength of Portland cements and properties of concretes as well as the level of Portland cement replacement without the reduction of compressive strength in comparison with the control cement and concrete without SCMs and to study the behavior of reinforcing steel bars in concretes incorporating such recycling waste.

## 2. Materials and Methods

Portland cement CEM I 42.5 (PRJSC Dyckerhoff Cement Ukraine, Zdolbuniv, Ukraine), ground granulated blast-furnace slag (ArcelorMittal Kryvyi Rih, Kryvyi Rih, Ukraine), and unactivated fly ash (Class F) derived from the Burshtyn power plant (Zakhidenergo, Halych district, 6 kilometres south-east from Burshtyn, Ivano-Frankivsk region, Ukraine) were used in this study as supplementary cementitious materials. The specific surfaces of GGBFS and unactivated FA were 310, 380, 510 and 310, 510 m^2^/kg, respectively. The commercially available lignosulfonate based plasticizer (PC “Zastava”, Chervonograd, Ukraine) with a specific gravity of 1.18 and solid content of 35% was used in the research. The properties of Portland cement are presented in Table 1.

The Portland cements of the second type CEM II, especially CEM II/A-S, is very popular in the construction market. The content of additions can range between 6–20% by mass in this cement according to EN 197-1. This is why the maximum amount of Portland cement (20% by mass) was replaced with GGBFS in the first stage of the research.

The properties of Portland cements and aggregates were determined according to Ukrainian standards [43,44,45,46,47]. The results of the tests of the aggregates are reported in Table 2.

The specific surface of Portland cement, GGBFS and FA was determined using the Tovarov device (Engineering firm "Integral", Podolsk, Russia). The Vicat apparatus ( PC “Biomedservice”, Poltava, Ukraine) was used to determine the setting time. The specific surfaces of GGBFS and fly ashes and residues in sieve 008 (the mesh opening size was 0.08 mm) are shown in Table 3.

**Figure 1:**
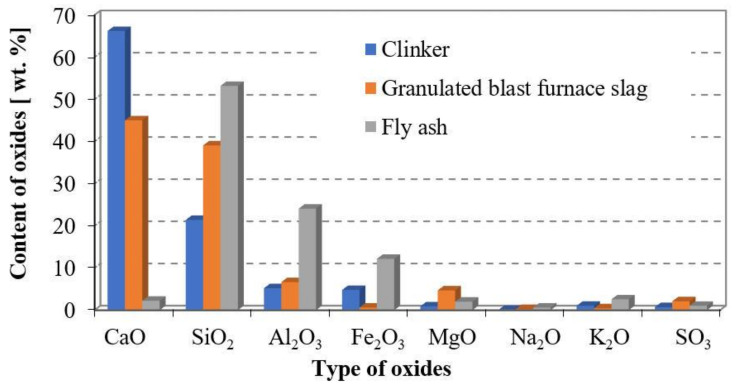
Chemical composition of clinker, granulated blast-furnace slag, fly ash.

The chemical composition of Portland cement, ground granulated blast-furnace slag and fly ash, which was determined by x-ray spectrometer ARL 9800 XP (Thermo Fisher Scientific, Waltham, MA, USA), is shown in Figure 1.

Fly ash in comparison with Portland cement and GGBFS is characterized by a higher amount of silica, alumina, and iron oxides, which provide its pozzolanic activity.

The morphology and chemical composition of the main mineral components of the studied materials were determined using scanning electron microscope SEM FEI Quanta 250 FEG, equipped with EDS (FEI Company, Hillsboro, OR, USA).

The mortar (cement:sand = 1:3, W/C = 0.39) was prepared, and three prisms 40 × 40 × 160 mm^3^ were molded according to [45] to determine the compressive strength of Portland cement (Table 1) and each Portland cement composition (Table 3). The test specimens in the mold had been left for at least 24 ± 1 h at a temperature of (20 ± 1) °C and relative humidity ≥ 90%, protected against shock, vibration, and dehydration. After removal from the mold, the test specimens were cured until the test in water at a temperature of (20 ± 1) °C. The results of the mortar tests are the mean value of six specimens.

Concrete mixes were designed according to [48]. Compressive strength of concrete was determined according to [49]. The 100 mm cubes were molded for each mixture proportion (Table 4). The test specimens in the mold have been left for at least 24 h at a temperature of (20 ± 5) °C, protected against dehydration, shock, and vibration. After removal from the mold, the test specimens were cured until test at a temperature of (20 ± 3) °C and relative humidity of (95 ± 5) %. The reported results are the mean value of three specimens. The standard deviation of obtained results was 0.6–1.5 MPa.

The water absorption and the freeze–thaw resistance of concretes were determined according to [50] and [51], respectively. The water absorption test was carried out in 28 days of concrete hardening. Three specimens of each concrete composition were dried in an oven to constant mass, then placed in a desiccator to cool and were finally weighed. Then, specimens were immersed in water at the temperature of 18–22 °C and stored there until full saturation.

Twelve specimens (six basic and six control specimens) of each concrete composition were molded and cured 28 days according to the Ukrainian standard (at the temperature of 20 ± 3 °C and the relative humidity of 95 ± 5 °C) to determine the freeze–thaw resistance of concretes. Then, the accelerated method was used according to [51] to determine the freeze–thaw resistance of concretes. Specimens cured 28 days at the temperature of 20 ± 3 °C and the relative humidity of 95 ± 5 °C were immersed in a 5% solution of sodium chloride to the 1/3 of their height and stored 24 h, then to 2/3 of their height and stored 24 h, and then the specimens were immersed in the solution completely and stored for 48 h. Then, all specimens were removed from the solution and the compressive strength of control specimens was determined before the freeze–thaw test of the basic specimens. The basic specimens were placed in a freezing chamber (HS280/75) and were frozen gradually up to −50 °C. The basic specimens were subjected to five freeze-thaw cycles. According to [52], five cycles of freeze–thaw in such a condition correspond to 100 freeze–thaw cycles in water at −20 °C [52]. After five freeze–thaw cycles, the compressive strength of the basic specimens was determined. Then, the compressive strength reduction was calculated.

The effect of Portland cement replacement with 10% by mass of GGBFS and 10% by mass of FA on the corrosion susceptibility of reinforcing steel in concrete was studied by the potentiostatic electro-chemical test according to [53]. Concrete specimens were prepared according to the recommendations in Annex A of the above-mentioned standard. The maximum size of coarse aggregate was 10 mm. Two mixtures were prepared: a reference control fine-grained concrete without SCMs (RCC) and a reference test one (RTC), which contained 10% by mass of GGBFS and 10% by mass of FA. The following mix-proportion was used: C = 350 kg/m^3^, S = 800 kg/m^3^, G (5–10 mm) = 1150 kg/m^3^, W = 175 kg/m^3^, plasticizer (0,9% by mass of a cement). The consistency class of fine-grained concrete mix was S1. Twenty percent by mass of Portland cement was replaced with 10% by mass of GGBFS and 10% by mass of FA in a reference test fine-grained concrete. Nine similar specimens (prisms of 40 × 40 × 160 mm^3^) of each mixture were molded. A smooth steel bar (working electrode) with the diameter of 6 mm and length of 120 mm was placed in the center of the mold and secured to prevent movement during filling and compaction. Before making the specimens, steel bars were cleaned using emery paper and then degreased with acetone. All specimens were cured 28 days at the temperature of 20 ± 3 °C and relative humidity of 95 ± 5 °C. Three specimens were tested after 28 days of hardening in the above-mentioned conditions. The other six specimens of each mix-proportion were kept in conditions of daily alternating wetting and drying (3 h of total immersion in water and 21 h in the conditions, where the test was conducted). Three specimens were tested after three and six months. Before measurement, specimens were saturated with drinking water by boiling them for 3 h. Before the beginning of the test, concrete was chipped from one end of the beam specimen, exposing the reinforcing bar by 20 mm ± 10 mm. Then the working, reference, and counter electrodes were connected to the polarization circuit. Measurements of the magnitude of the current in microamperes were carried out in concrete specimens in (60 ± 5) min after turning on the potentiostat. The output of the potentiostat was raised, and the current recorded when passing the range from the steady-state potential to plus 1000 mV for 60 min. For each test specimen, the surface area of the working electrode, which was in direct contact with the concrete, was calculated. Using the calculated area of the rebar, the current density was determined.

## 3. Experimental Results and Discussions

The strength of building materials is an important characteristic, which defines their quality. This study focused mainly on the influence of SCMs on the compressive strength of Portland cements and properties of concretes as well as the corrosion susceptibility of reinforcing steel bars. It is a well-known fact that there are two schemes that show how GGBFS can be added to Portland cement. It can be mixed with constituents of cement by intergrinding or after previous separate grinding to the appropriate specific surface. The second way was used as it is more effective. It allows for the achievement of a more complete realization of potential hydraulic properties of GGBFS, because the size of the slag particles remained rather coarse after intergrinding of clinker and GGBFS due to the poor grindability of the GGBFS. Therefore, the Portland cement and GGBFS, ground to the appropriate specific surface previously, were mixed in the proportions that are shown in Table 3.

**Figure 2:**
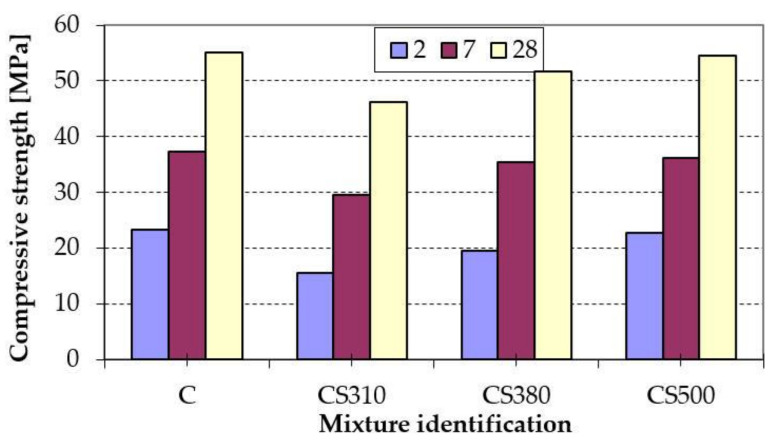
Compressive strength of Portland cements.

As can be seen from Figure 2, the fineness of GGBFS had a significant influence on the compressive strength of a cement. The compressive strength of mortar (CS310) on the basis of Portland cement containing 20% by mass of GGBFS with a specific surface of 310 m^2^/kg was 34, 21 and 16% lower than the control mortar (C) after 2, 7, and 28 days of hardening, respectively. The increase of fineness of GGBFS caused gradual growth of strength at both early and later ages in comparison with CS310. The compressive strength of mortar CS380 was reduced by 6% after 28 days of hardening, while CS500 almost reached the compressive strength of the control mortar. However, GGBFS with a specific surface of 380 m^2^/kg was used for further research, because the process of slag grinding up to 500 m^2^/kg would be a rather expensive and simultaneously time consuming process. Moreover, GGBRS is becoming more and more expensive on the market year by year, and scientists are looking for SCMs to replace it.

**Figure 3:**
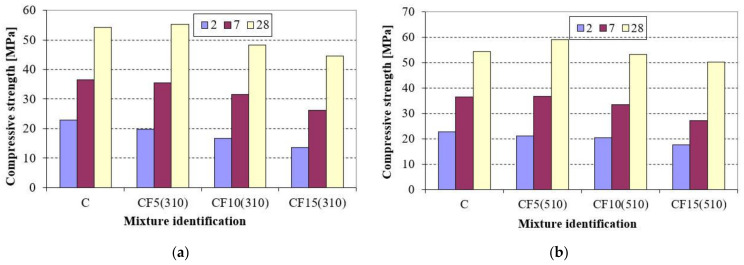
Compressive strength of Portland cements incorporating fly ash with specific surfaces of 310 m2/kg (**a**) and 510 m^2^/kg (**b**).

Widespread by-products such as fly ash, which needs to be utilized, were used to improve the properties of cements. The specific surface of fly ash can be different and range widely at the thermal power plant. The unactivated fly ash with specific surfaces of 310 m^2^/kg and 510 m^2^/kg was used in the research. The Portland cement CEM I 42.5 was replaced with 5% (CF5(310), CF5(510)), 10% (CF10(310), CF10(510)) and 15% (CF15(310), CF15(510)) by mass of FA. The obtained results are presented in Figure 3.

A reduction in compressive strength was observed in mortars CF10(310) and CF15(310). Nicoara et al. [13] observed the same tendency. It was also found by Sanytsky et al. [20] that the replacement level of Portland cement with fly ash was increased due to its mechanical activation up to 40% by mass, but the strength reduction took place at all ages in comparison with activated Portland cement. It should be noted that the compressive strength of mortar CF5(310) decreased at the early age (two and seven days), but the growth of the kinetics of strength development was observed later, and it even slightly exceeded the compressive strength of the control mortar C at 28 days. The substitution levels of Portland cement for fly ash can be different. This depends on the specific surface of fly ash, because the size of the particles is very important. The reaction takes place on the surface and as a result, finer materials react faster [54,55]. The fly ash with a specific surface of 310 m^2^/kg was rather coarse and 5% by mass of Portland cement could only be replaced without the compressive strength reduction. At the same time, the usage of fly ash with a higher specific surface (510 m^2^/kg) made it possible to increase the replacement level of Portland cement without the reduction of strength after 28 days. The results in Figure 3 indicate that the tendency remained the same when the fly ash with the specific surface of 510 m^2^/kg was used, but the replacement level could be increased to 10% by mass.

**Figure 4:**
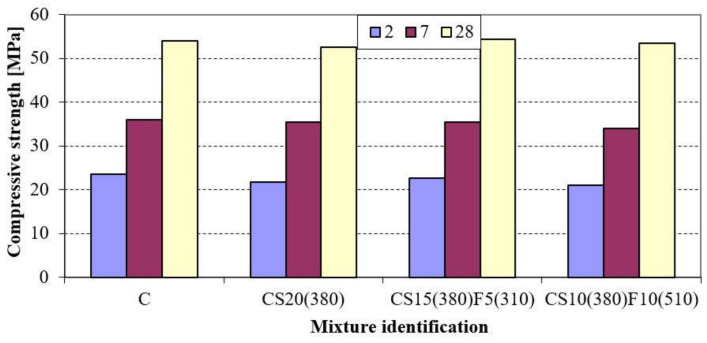
Compressive strength of Portland cements.

The literature review and analysis of the obtained results show the necessity of combining GGBFS and FA. Compressive strength of mortars containing 10 and 15% by mass of GGBFS as well as 5 and 10% by mass of FA with specific surfaces of 310 (CS15(380)F5(310)) and 510 m^2^/kg (CS10(380)F10(510)) was studied. The mortar incorporating 20% by mass of GGBFS (CS20(380)) was used for comparison. The above-mentioned mortars containing the same amount of SCMs (20% by mass) was studied and compared with control mortar C. The results in Figure 4 indicate that mechanical strength of mortar containing 20% by mass of GGBFS is lower in comparison with control concrete.

However, the kinetics of the strength gain of mortars in which Portland cement was replaced with both GGBFS and fly ash was lower for up to two days, but due to the synergetic effect of the combination of these two by-products, accelerated, leveled CS10(380)F10(510), and even exceeded CS15(380)F5(310) in the compressive strength of control mortar at later ages (28 days). The existence of this effect was also confirmed by many researchers [15,20,34,55].

**Figure 5:**
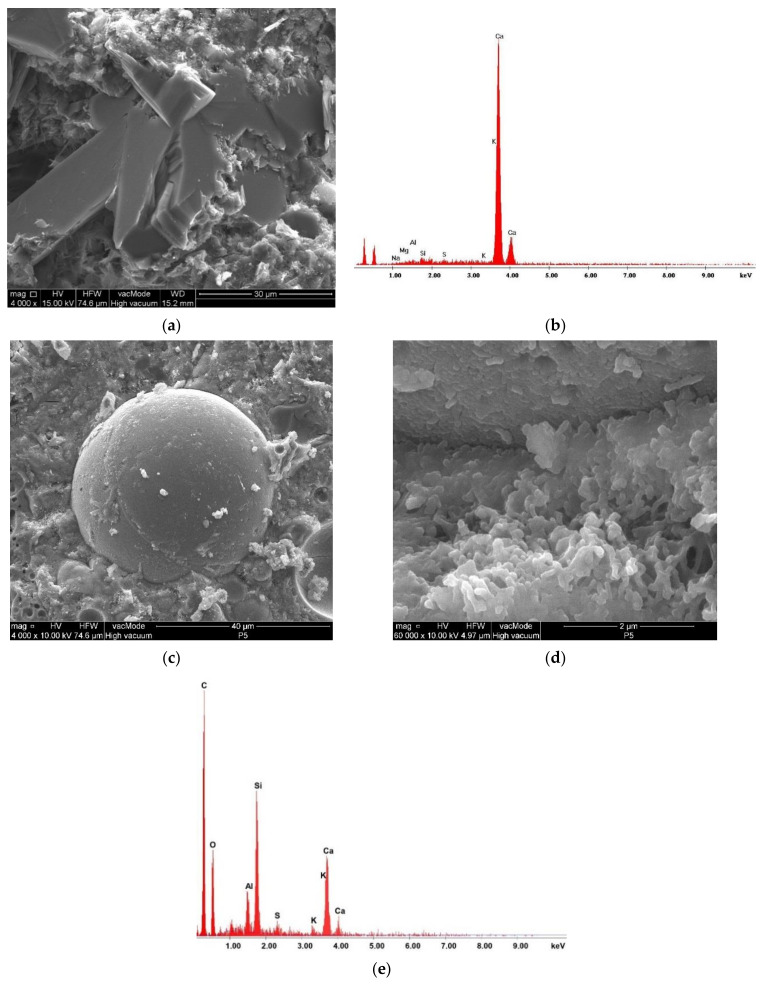
Microstructure and EDS spectra of Portland cement paste: (**a**,**b**) without SCMs; (**c**–**e**) containing 15% by mass of GGBFS and 5% by mass of FA.

Scanning electron microscopy was used to study the peculiarities of microstructure formation of cement paste without SCMs and paste incorporating 15% by mass of GGBFS and 5% by mass of fly ash. As shown in Figure 5a, many hydration products are formed. The plate-like portlandite is traditionally observed in the pure cement paste without SCMs as a result of the reactions between main minerals of clinker such as alite and belite and water, which was confirmed by the EDS spectra (Figure 5b). The structure of cement paste incorporating GGBFS and fly ash became denser due to the formation of an additional amount of fiber-like crystals of hydrosilicates, ettringite needles, and hydroaluminosilicates in the non-clinker part, which was confirmed by the results of microprobe analysis (Figure 5e), because GGBFS and FA are the source of active Al_2_O_3_ and SiO_2_. The above-mentioned hydration products appear in the vacant space of cement matrix and also on the fly ash surface (Figure 5c). Unactivated fly ash also optimizes the particle size distribution in the binding system and provides the prolonged time of cement hydration, which results in obtaining a more refined and compact structure over time. The colmatation of pores and the growth of the cement paste strength take place as a result (Figure 5d).

GGBFS and FA are also used successfully in the technology of concrete production as cement replacements. In this work, a concrete mix design was carried out. Concrete mixture proportions are presented in Table 4. The slump of the mixtures was maintained in the range of 190–200 mm.

**Figure 6:**
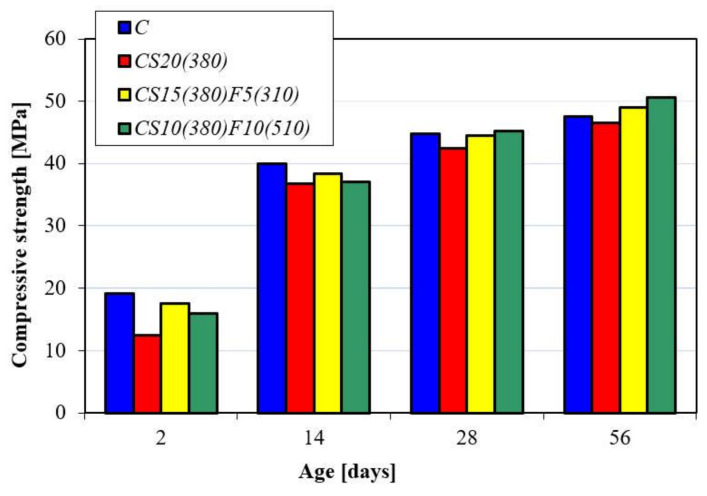
Compressive strength of concretes.

As shown in Table 4, the replacement of 20% by mass of Portland cement with GGBFS (310) resulted in the reduction of compressive strength by 34.9%. The targeted workability was obtained at the same W/C ratio. Concrete incorporating 15% by mass of GGBFS and 5% by mass of fly ash (310) had better workability because the spherical particles of FA produced the ball bearing effect. It allows the designed slump at lower W/C ratio to be obtained, which was equal to 0.55. When the content of FA was increased from 5% (F5(310)) to 10% (F10(510)) by mass and the amount of GGBFS (380) was decreased from 15% to 10% by mass, the W/C ration of the concrete decreased from 0.59 to 0.55, causing a slight increase in compressive strength after two days of hardening in comparison with concrete containing 20% by mass of GGBFS (380). It should be noted that the percentages of compressive strength reduction of concretes containing GGBFS and FA reduced after 14 days, then leveled and even exceeded the strength of C and CS20(380) after 56 days of hardening. Ghais et al. [30] also confirmed that the addition of 10% by mass of fly ash increased the concrete strength. Tan et al. [32] concluded that the compressive strength of concrete incorporating 10% by mass of finely ground fly ash and 10% by mass of finely ground granulated blast furnace slag increased at all ages. Better particle packing takes place and a denser structure is formed, resulting in an improvement in the long-term compressive strength and the highest strength (50.6 MPa) of concrete CS10(380)F10(510) at 56 days of hardening (Figure 6).

**Figure 7:**
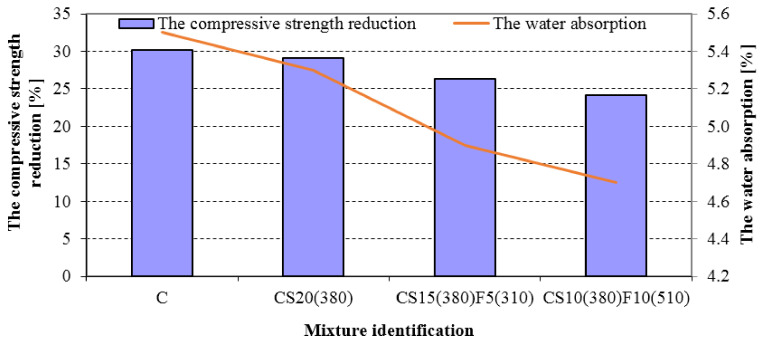
The water absorption and compressive strength reduction of concretes after 100 freezing/thawing cycles.

The water absorption confirmed the positive effect of the rational selection of SCMs and their combination including the individual properties of all constituents to obtain a synergetic effect. Figure 7 shows that concretes containing GGBFS and FA had the lowest water absorption due to the tighter microstructure.

The freeze–thaw resistance of concretes after 100 freezing/thawing cycles was also investigated because it defines the durability of concretes. If concrete in a water-saturated state is exposed to the alternate freezing and thawing, the water freezes and expands by about 9%. The absence of enough space to accommodate this extra volume results in a disruptive pressure, which will be created. If the internal stresses exceed the tensile strength of concrete, they can eventually cause its cracking, scaling, and crumbling [56,57]. The hydraulic and osmotic pressure will cause the further increase of the disruption. If the water freezes in the completely water filled pores, the unfrozen water has to be transported to empty spaces through the capillary and gel pores of the cement paste, creating a hydraulic pressure, which depends on the distance between the pores and the fineness of the capillary pores. A longer distance and finer pores result in the pressure increase, which will be further enhanced by osmotic pressure causing the external and internal damage of concrete [58].

The compressive strength reduction of designed concretes is reported in Figure 7. As can be seen, the compressive strength reduction of concretes decreased gradually from 30.2 for control concrete to 24.1% for concrete incorporating 10% by mass of GGBFS (380) and 10% by mass of FA(510) after 100 freezing/thawing cycles. Łukowski et al. [59] observed some worsening of the frost resistance of concrete incorporating slag, but it is not clear from their study what the specific surface of GGBS was. Lindvall et al. [60] reported that concrete with pulverized fuel ash (PFA) has similar frost resistance to CEM I concrete if the maximum content of PFA is 25% by mass and (w/c)_eq_ ≤ 0.45. The higher resistance of concrete CS10(380)F10(510) to freezing and thawing damage is obtained due to the improvement of the pore structure. The higher amount of secondary CSH gel obtained due to the pozzolanic reaction results in a decrease in the capillary porosity of concrete incorporating GGBFS and FA, which confirms the efficiency of the simultaneous combination of these SCMs.

**Figure 8:**
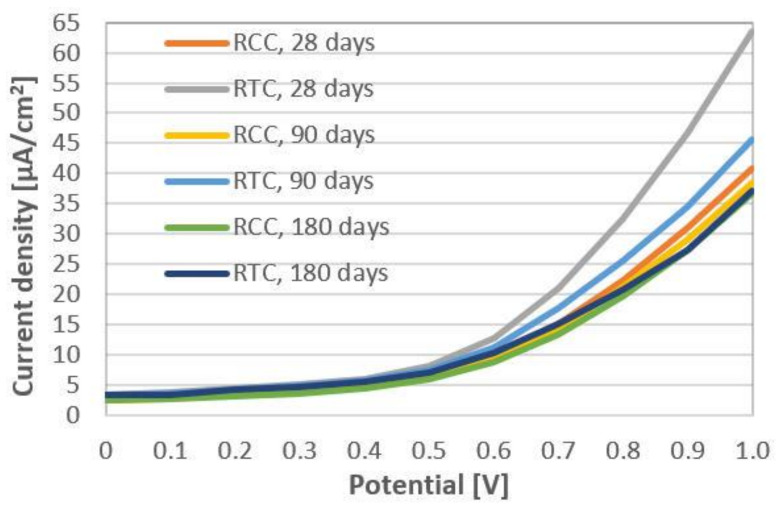
The current density of the working electrode surface in concretes at 28, 90, and 180 days of hardening.

The replacement of cement with SCMs in concretes also results in the reduction of the clinker and as a result, calcium hydroxide, which is produced during Portland cement hydration and is responsible for creating a protective passive layer on the rebars due to the alkaline environment. This is why the corrosion susceptibility of reinforcing steel bars in such concretes was studied. As can be seen from Figure 8, the deference between the current density of the working electrode surface was very small up to 0.5 V. If the output of the potentiostat is raised to values that are higher than 0.5 V, the corrosion current density increased more for concrete containing 10% by mass of GGBFS (380) and 10% by mass of FA (510) after 28 days of hardening in comparison with the reference control fine-grained concrete. The current density was 40.9 and 63.5 µA/cm^2^ at 1 V for RCC and RTC, respectively. The content of calcium hydroxide in RCC was lower because 20% by mass of Portland cement was replaced with SCMs and the pozzolanic reaction also consumed some amount of Ca(OH)_2_, reducing its content in hardened concrete, and likely a thinner protective passive layer formed on the rebars. Over time, the current density deference of compared concretes was reduced to 7.1 µA/cm^2^ after 90 days and even almost levels after 180 days at a potential of 1 V due to the formation of the tighter structure of concretes incorporating GGBFS and FA. Polder [42] also observed the decrease in the risk of the steel reinforcing bar corrosion in such a binding system, even in chloride contaminated environments.

## 4. Conclusions

The experimental work was done to evaluate the influence of a granulated blast-furnace slag and fly ash on the properties of Portland cements and concretes as well as the corrosion susceptibility of the reinforcing steel bars. The above-mentioned recycling waste was used for a partial replacement of binding materials in this article. The following conclusions can be drawn from the study:

- The increase of fineness of GGBFS from 310 to 500 m^2^/kg resulted in the growth of Portland cement strength by 47 and 18% at 2 and 28 days of hardening, respectively.
- The kinetics of strength development of Portland cements and concretes incorporating GGBFS and FA is lower at the early age of structure formation, but accelerates at later ages due to the pozzolanic reaction that takes place.
- The synergetic effect of the compressive strength development was revealed in Portland cement and concrete containing granulated blast-furnace slag and fly ash.
- The replacement level of clinker in cements and Portland cement in concretes with GGBFS and FA depends on their specific surface.
- The obtained results show the efficiency of unactivated fly ash usage for the cement and concrete production and the necessity of permanent control of fly ash fineness to determine the replacement level of the clinker in a cement and a cement in a concrete.
- Simultaneous use of supplementary cementitious materials of different nature activity such as GGBFS (latent hydraulic) and FA (pozzolanic) allows for the improvement in the microstructure of concretes due to the formation of an extra amount of ettringite and tobermorite-like low-basic calcium hydrosilicates as well as hydroaluminosilicates in the non-clinker part of cement paste. Unactivated fly ash also allows the particle size distribution to be optimized in cements and concretes to archive the prolong time of cement hydration, which results in obtaining a more refined and compact structure over time due to its role both as a mineral addition with pozzolanic properties and as a microfiller. As a result, the durability and the stability of rebars in such concretes increase, which conforms to the strategy of sustainable development in the construction.
- Incorporation of cement replacement materials such as GGBFS and FA in concretes results in the reduction of water absorption from 5.5% to 4.7% and, as a result, in the increase of resistance to freezing and thawing damage, improving its durability. The decrease in compressive strength reduction was observed from 30.2 for concrete C to 24.1% for concrete CS10(380)F10(510) after 100 freeze–thaw cycles.
- Concretes containing 20% by mass of SCMs will have a better corrosion resistance in comparison with the concrete on the basis of Portland cement CEM I. Steel reinforcement protection does not worsen in concretes incorporating 10% by mass of GGBFS and 10% by mass of FA due to the low Portland cement substitution rate and their tighter microstructure. The obtained results concerning the corrosion susceptibility of reinforcing steel bars in concrete incorporating GGBFS and FA will be taken into account in the future developments of this study in the real reinforced concrete elements.

## Figures and Tables

**Table 1:** Physical and mechanical properties of Portland cement.

**Specific Surface [m^2^/kg]**	**Residue on Sieve 008** **[%]**	**Water Demand** **[%]**	**Setting Time [min]**		**Compressive Strength [MPa]**	
			Initial	Final	2 days	28 days
390	2.8	29.0	150	240	29.5	53.5

**Table 2:** Properties of aggregates.

Aggregate Type	Density [g/cm^3^]	Bulk Density [kg/m^3^]	Voidage [%]	Dust and Clay Particles [%]	Water Absorption [%]	Fineness Modulus
Fine (quartz sand)	2.65	1438	45.7	0.4	-	1.85
Coarse (granite gravel, 5–20 mm)	2.68	1370	48.9	0.3	0.6	-

**Table 3:** Composition of Portland cements and the fineness of SCMs.

Mixture Identification	Portland Cement [% by Mass]	GGBFS [% by Mass]	FA [% by Mass]	Specific Surface, [m^2^/kg]		Residue on Sieve 008, [%]	
				GGBFS	FA	GGBFS	FA
C0	100	0	0	-	-	-	-
CS310	80	20	0	310	-	6.0	-
CS380	80	20	0	380	-	5.4	-
CS500	80	20	0	500	-	4.8	-
CF5(310)	95	0	5	-	310	-	14.4
CF5(510)	95	0	5	-	510	-	1.3
CF10(310)	90	0	10	-	310	-	14.4
CF10(510)	90	0	10	-	510	-	1.3
CF15(310)	85	0	15	-	310	-	14.4
CF15(510)	85	0	15	-	510	-	1.3
CS15(380)F5(310)	80	15	5	380	310	5.4	14.4
CS10(380)F10(510)	80	10	10	380	510	5.4	1.3

**Table 4:** Mixture proportions.

Mixture Identification	Portland Cement [kg/m^3^]	GGBFS [kg/m^3^]	GGBFS [%]	FA [kg/m^3^]	FA (%)	Plasticizer [% of Cement]	W/C
C	350	0	-	-	-	0.9	0.59
CS20(380)	280	70	20	-	-	0.9	0.59
CS15(380)F5(310)	280	52.5	15	17.5	5	0.9	0.55
CS10(380)F10(510)	280	35	10	35	10	0.9	0.53

Note: the following are valid for all mixtures: fine aggregate = 690 kg/m^3^ and coarse aggregate (5–20 mm) = 1150 kg/m^3^.

## Data Availability

The data presented in this study are available on request from the corresponding author.
